# Forensic autosomal and gonosomal short tandem repeat marker reference database for populations in Burkina Faso

**DOI:** 10.1038/s41598-024-58179-4

**Published:** 2024-03-28

**Authors:** Moutanou Modeste Judes Zeye, Serge Yannick Ouedraogo, Prosper Bado, Abdou Azaque Zoure, Florencia W. Djigma, Xiang Wu, Jacques Simpore

**Affiliations:** 1 Department of Medical Parasitology, School of Basic Medical Sciences, Central South University, No. 172, Tongzipo Road, Changsha, 410013 Hunan People’s Republic of China; 2 Department of Oncology, School of Clinical Medicine, Shandong Cancer Hospital, Shandong First Medical University, Shandong Academy of Medical Sciences, 6699 Qingdao Road, Huaiyin District, Jinan, 250000 Shandong People’s Republic of China; 3 Laboratory of Molecular Biology and Genetics (LMBG) (Labiogene), University Joseph KI-ZERBO, CERBA/LABIOGENE, 01, BP 364, Ouagadougou 01, Burkina Faso; 4 Human Evolution, Department of Organismal Biology, Evolutionary Biology Centre, Uppsala University, Uppsala, Sweden; 5 Department of Biomedical and Public Health, Research Institute of Health Sciences (IRSS/CNRST), 03 BP 7192, Ouagadougou 01, Burkina Faso

**Keywords:** Burkina Faso, Population data, Genetic structure, Forensic parameters, Autosomal STRs, X-chromosome STRs, Genetic variation, Genetic linkage study

## Abstract

Tandem repeat genetic profiles used in forensic applications varies between populations. Despite the diversity and security issues in the Sahel that require the identification of victims (soldiers and civilians), Burkina Faso (BF) remains understudied. To fill this information gap, 396 unrelated individuals from BF were genotyped using a MICROREADER 21 ID System kit. All 20 short tandem repeat (STR) loci tested passed the Hardy–Weinberg equilibrium (HWE) test. The combined powers of exclusion for duos (CPE duos) and trios (CPE trios) for the 20 tested loci were 0.9999998 and 0.9999307, respectively. The probability that two individuals would share the same DNA profiles among the BF population was 9.80898 × 10^–26^. For the X-chromosome STR analysis, 292 individuals were included in this study using a MICROREADER 19X Direct ID System kit. Among the 19 loci, no significant deviations from HWE test were observed in female samples after Bonferroni correction (p < 0.05/19 = 0.0026), except for loci GATA165B12 and DXS7423. The results showed that the combined power of exclusion (CPE) and the combined power of discrimination in females (CPDF) and males (CPDM) were 0.999999760893, 0.999999999992, and 1, respectively. Comparison with other African sub-populations showed that geographical proximity is a reliable indicator of genetic relatedness.

## Introduction

**Figure 1:**
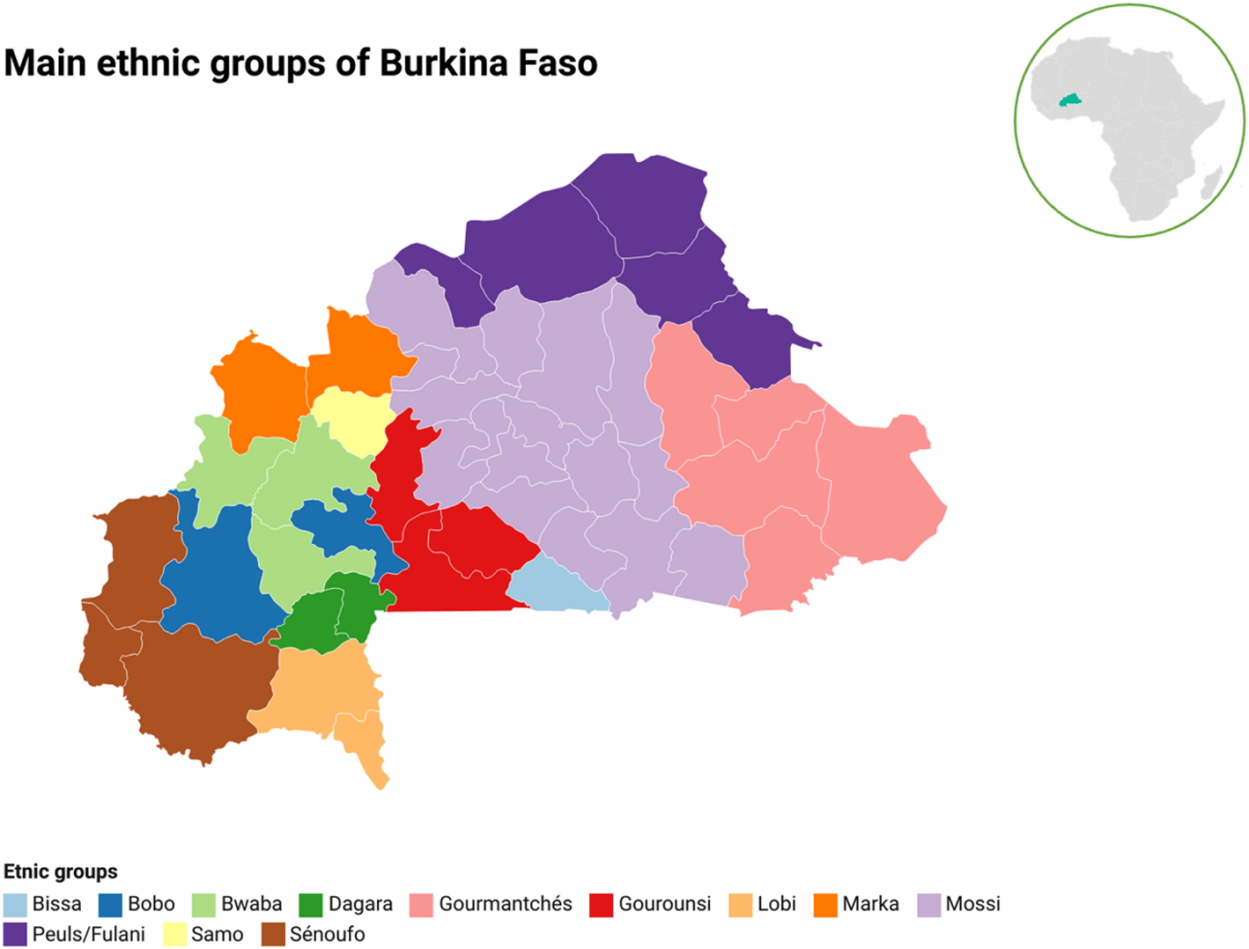
Geographic distribution of the main ethnic groups from Burkina Faso (Maps generated with DATAWRAPPER).

Burkina Faso (BF) is a country located in the middle of West Africa with 63 ethnic groups speaking local languages^1^. They belong to the Niger-Congo language phylum and are divided into five groups: Mande, Gur, Kru, West Atlantic, and Dogon^2^. Figure 1 presents a map of the main ethnic groups in BF. Despite a large population size, 20 million people reported in the last census^3^, data on genetic structure and forensic parameters based on autosomal STR (aSTR) and X-chromosome STRs (X-STRs) are still lacking. This genetic research gap exists while persistent security concerns in Sahel region^4^, necessitate effective measures to identify individuals and remains of deceased persons^5^. However, to assign legal significance to DNA profiles and conduct genetic studies and family searches, population data acquired from well-characterized populations are essential. Population genetic studies remain necessary in forensic science because they quantify the genetic variation observed within a population group or among different population groups, in terms of allele and genotype frequencies^6^.

To date, the use of aSTR for human identification remains the gold standard in forensic genetics^7^. The X-STRs on the other hand are generally used when the Y-chromosome (Y-STR) and aSTR fail to provide satisfactory results for complex kinship or paternity test cases. In fact, in some specific cases in forensic investigations, such as testing mother-son kinship, the use of X-STRs is more efficient^8^. The chance of exclusion in such cases is similar when X-STRs are used in father-daughter tests^9^. In father-daughter tests, a significant advantage of using X-STRs is that the male X chromosome is entirely transmitted to the female offspring^8^. Furthermore, in some cases of paternity testing, such as a test of a half-sister without the disputed father’s DNA or grandmother-granddaughter without the parent’s DNA, the use of X-STR is more informative than any other forensic genetic marker^10^.

Populations from BF has been the subject of limited research on forensics markers. To the best of our knowledge, only one study has been conducted on 29 Y-STR loci among ethnolinguistic groups in the BF^11^. The availability of forensic data is an important step in establishing human DNA identification systems^12^. These data are even more important in view of the current difficult security contexts of sub-Saharan countries, such as BF. Currently, the identification of human remains discovered as a result of armed conflicts, the search for missing persons, and the growing demand for paternity testing^13^ makes population data and forensic parameters useful information for strengthening judicial and security systems in particular, as well as for the scientific community in general^14^. This study contributes to the establishment of an STR allele reference frequency database for the BF population for forensic and population genetics studies. It also establishes the genetic affinities of the BF population to other reference populations worldwide and thereby contributes to both forensic and bioanthropological research.

## Results

### aSTR

#### Forensic statistics and dataset description

We successfully typed 396 DNA samples collected from self-declared, unrelated male and female volunteers. The 20 autosomal STRs typed in this study yielded unique DNA profiles for each volunteer within the BF population, i.e. no participant shared identical profiles. The distribution of allele frequencies and forensic statistical parameters of the BF population is shown in the supplementary material (Table S1). Based on these results, 281 alleles were identified. In the overall dataset, Locus FGA showed 28 different alleles, which had the highest number of alleles in our dataset (Table S1). Loci D13S317, D7S820, and TPOX showed the lowest number of alleles, with eight alleles. The MP and PIC values ranged from 0.0189 (Penta E) to 0.1512 (TPOX) and from 0.6388 (D13S317) to 0.8957 (Penta E), respectively. The highest PD and GD values for the PentaE locus were 0.9811 and 0.9044, respectively. Locus D13S317 showed the lowest PD (0.8537) and GD (0.6921) values.

We investigated the Power of Exclusion for “duos” involving two persons, for “trios” involving three persons (Table S2). We computed the real combined power of exclusion (CPE) for “duos” and found 0.999999837, while the CPE value was 0.999930699 for the “trios”. The typical Paternity Index (TPI) displayed in the (Table S2) is for random non-excluded men at a given locus.

#### Hardy Weinberg equilibrium

No locus deviated from HWE after Bonferroni correction (P 0.05/21 ≈ 0.0023) (Table S3). When comparing male and female samples using ARLEQUIN v3.5 software. There was no statistically significant variation in the distributions of allele frequencies (p > 0.05).

#### The genetic structure between ethnic groups from BF and published data

**Figure 2:**
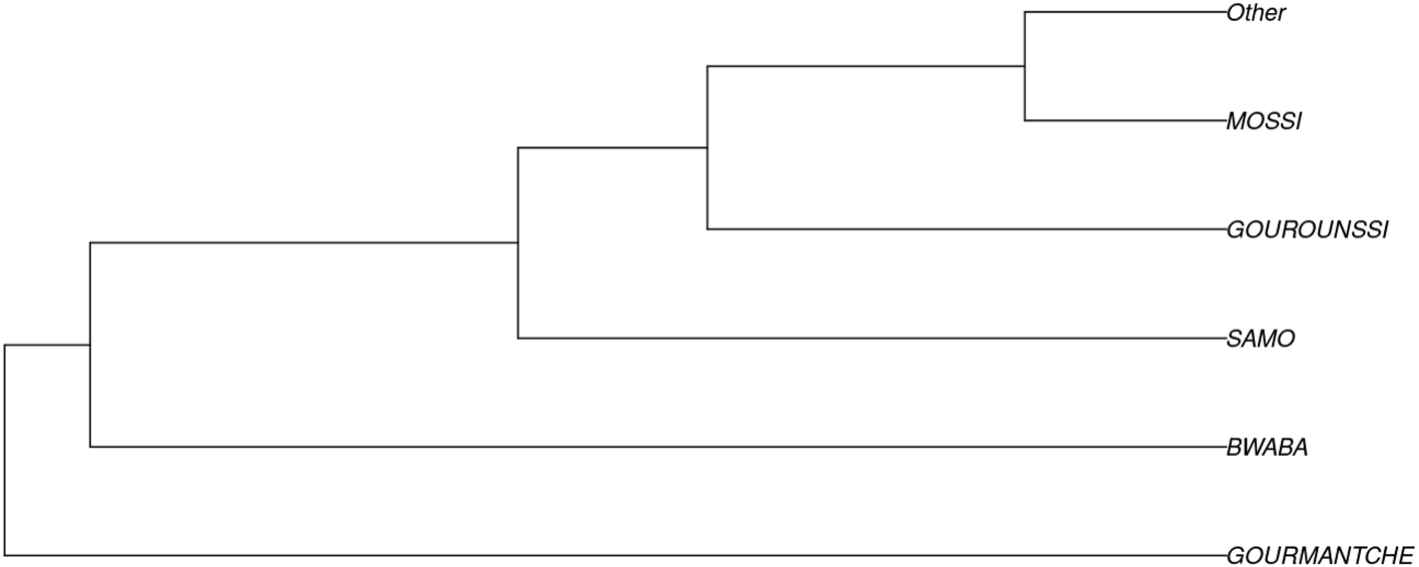
Phylogenetic tree of Burkina Faso ethnic groups involved in the current study. For statistical reasons ethnic groups involved in the current study with participants under 20 individuals have been grouped under “Other” (generated using STR Analysis for Forensics software (STRAF) version 2.1.5^34^).

Pairwise F_ST_ genetic distance analysis based on the 21 autosomal STRs investigated in this study showed low or no significant differentiation between the 25 BF ethnic groups listed in this study (Table S4) and the population tree displayed in Fig. 2. This may be attributed to the increasing trend of people marrying beyond their ethnic boundaries.

**Figure 3:**
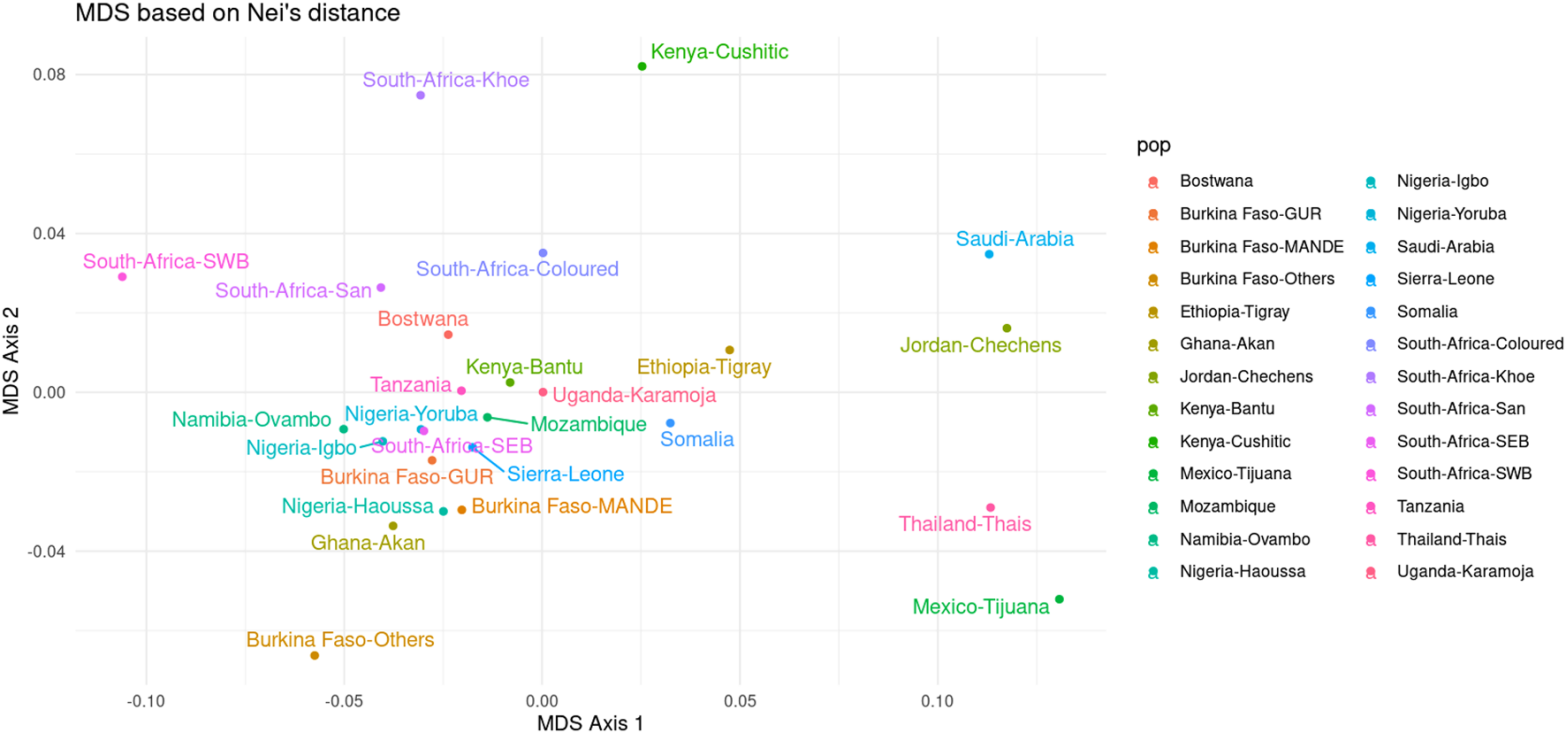
MDS based on Nei's distance between Burkina Faso and 23 populations from published data, mainly in Africa. *NB: Fourteen loci commonly used to perform MDS based on Nei’s distance were D19S433, D5S818, D21S11, D18S51, D3S1358, D13S317, D7S820, D16S539, CSF1PO, D8S1179, TPOX, TH01, D2S1338, and FGA* (generated using STR Analysis for Forensics software (STRAF) version 2.1.5^34^).

The allelic frequencies (Table S5) of some loci in this study were compared with those of 23 population groups, mainly living in Africa (19/23), from published data (Table S4). A total of 5432 individual samples were investigated. West African populations speaking Niger Congo languages, or populations with west African ancestry from other parts of Africa e.g. Bantu-speakers from Kenya, Mozambique, South Africa and Namibia, all formed part of a main central cluster, indicating their similar west African, Niger Congo associated ancestry. Non-African populations separated out towards the left side of the MDS plot and East African populations that showed substantial non-African admixture in previous studies (e.g. populations from Somalia and Ethiopia)^15^ are located in-between the west African cluster and the non-African populations. Populations with East African ancestry (Kenya-Cushitic) and southern African Khoisan ancestry (Khoe, San and Coloured) separated out towards the top of the plot (Fig. 3). Interestingly the BF-Others group separate from the west African cluster towards the bottom of the plot.

### X-STR

#### X-STR haplotype diversity and allelic frequencies

We successfully typed 292 DNA samples from 123 unrelated females and 169 males from the Burkina Faso population. The 19 X-STR loci investigated in this study were suitable for the BF population as none of the individuals involved in this study shared the same genotype profile. The genotype profiles (raw data) are available from the authors upon request.

The distribution of allele frequencies is shown in Table S6 for the pool (male and female samples), in Table S7 for the female samples, and in Table S8 for the male samples. Among the allele frequencies in the pooled dataset, we encountered 255 different alleles, and the allele numbers ranged from six at GATA165B12 to 43 at DXS10135. Among the 255 alleles observed, allele frequencies ranged from 0.0024 to 0.7482. However, 198 different alleles were found when considering the allele frequencies in the female dataset, with the lowest number of five alleles at DXS6810 and GATA165B12, and the highest number of 36 alleles found at DXS10135 (Table S7). A total of 226 alleles were observed in the male sample, ranging from five at the GATA165B12 locus to 35 at the DXS10135 locus (Table S8). In general, DXS10135 displayed the highest polymorphism among the studied 19 X-STR loci.

#### Forensic parameters of 19 X-STR loci among Burkina Faso ethnolinguistic group

The statistical parameters of forensic interest for the 19 X-STRs, based on pooled allele frequencies, are shown in Table S6. Two of the 169 analysed male samples showed locus dropout, including one for locus DXS689 and one for locus DXS10135. These two samples were excluded from the statistical analyses. Similar results have been reported by Bini et al.^16^. However, in this study, dropout was found at loci DXS10148 and DXS10146, instead of DXS689 and DXS10135. Bini et al. suggested that: “these silent alleles are due to one or more mutations in the primer binding sites, which reduces the efficiency of the PCR reaction, as previously observed in African populations”. Further investigation is required before any conclusions can be drawn.

Regarding forensic parameters, locus DXS10135 (which had the highest allele number with 43 unique alleles) was the most informative locus, with the highest value for all efficient forensic parameters computed in this study. This result is in agreement with previous studies on population data based on 19 X-STR loci carried out by He et al.^17^ in the Sichuan Tibetan minority ethnicity group, Xiao et al.^18^ from the Guangdong Han population, Jia et al.^19^ in Beijing Han individuals, and Lin et al.^20^ in the Sierra Leone population from Freetown, where the locus DXS10135 was the most informative locus with 20, 21, 27, and 60 unique alleles, respectively. In contrast, the locus GATA165B12 (with six alleles) demonstrated the lowest polymorphism and was detected as the lowest value for all forensic-efficiency parameters. Compared with previously published data, our results are different. The lowest informative loci were: DXS7423 (4 alleles) for He et al.^17^, Xiao et al.^18^, and DXS6800 for Jia et al.^19^, DXS6807 (8 alleles) for Lin et al.^20^. This suggests that forensic statistical parameters should be computed for each population before establishing a panel of loci for forensic casework. We believe that this will allow us to set up a suitable panel of X-STR loci for a specific population, in which the selected loci will retain the most polymorphic and informative loci.

The combined power of exclusion (CPE), the combined PDM, PDF, as well, as the combined power of MECKrüger, MECKishida, MECDesmarais, and MECDesmarais Duo were 0.999999760892967, 0.999999999992047, 1, 0.999999930181708, 0.999999999990194, 0.999999999990369, and 0.999999983891293 respectively. The cumulative CPE, CPDM, CPDF, MECKrüger, MECKishida, MECDesmarais, and MECDesmarais Duo values were > 0.999999. This suggests that the 19 X-STR loci involved in this study could be used in paternity test casework, particularly also for paternity cases in BF.

#### Linkage groups and linkage disequilibrium analyses

This study represents the first study that investigates population data and relevant forensic parameters based on the X-STR loci in BF populations. Consequently, no information regarding any linkage group was discovered during the investigation of X-STRs in the BF population for comparison. Furthermore, the MICROREADER 19X Direct ID System kit used in this study is different from other commercially available kits. The results showed that there was no LG in this panel of 19 X-STRs. This was mentioned by the manufacturer^18^. The reason for this choice is to allow computation of the likelihood ratio without considering any linkage groups. The advantage of the lack of LG in this kit is that it can upgrade the combined polymorphism and diversity index and maximise the CPD and CMEC.

The exact test of linkage disequilibrium (LD) between all the 171 pairs of loci in male samples of Burkina Faso (Table S9) revealed a significant p-value with p < 0.0001 (p-value = 0.05/169 = 0.00029 after Bonferroni's correction) for 8 pairs of loci, the concerning numbers are in bold (Table S9). The eight pairs of loci were DXS6795/GATA172D05, DXS6803/GATA172D05, DXS6807/GATA172D05, DXS9907/DXS7133, GATA172D05/DXS101, GATA172D05/DXS6800, DXS101/DXS6800, and DXS7133/DXS6800.

On the other hand, the LD computed between all pairs of loci in the female sample is presented in Table S10. The results revealed that among the 171 pairs of loci compared, three pairwise pairs had a p-value < 0.05 (see Table S10, the numbers in bold). However, after Bonferroni correction, no LD was found, with a significance level of 0.00029 (p = 0.05/171).

#### The Hardy–Weinberg equilibrium (HWE) in the female sample

No significant deviation from the HWE was observed after Bonferroni correction (the critical HWE p-values were 0.0026 (p < 0.05/19 = 0.0026), except for the loci GATA165B12 and DXS7423. Our results revealed a significance level of 0.00029 (p = 0.05/169) after the Bonferroni correction for the male sample. This result may be interpreted as random association between alleles at different loci in the Burkina Faso ethnic group. This finding is similar to that of a study conducted on the Sierra Leone population in Freetown^20^.

#### Genetic Structure between BF ethnolinguistic groups based on 19 X-STRs

**Figure 4:**
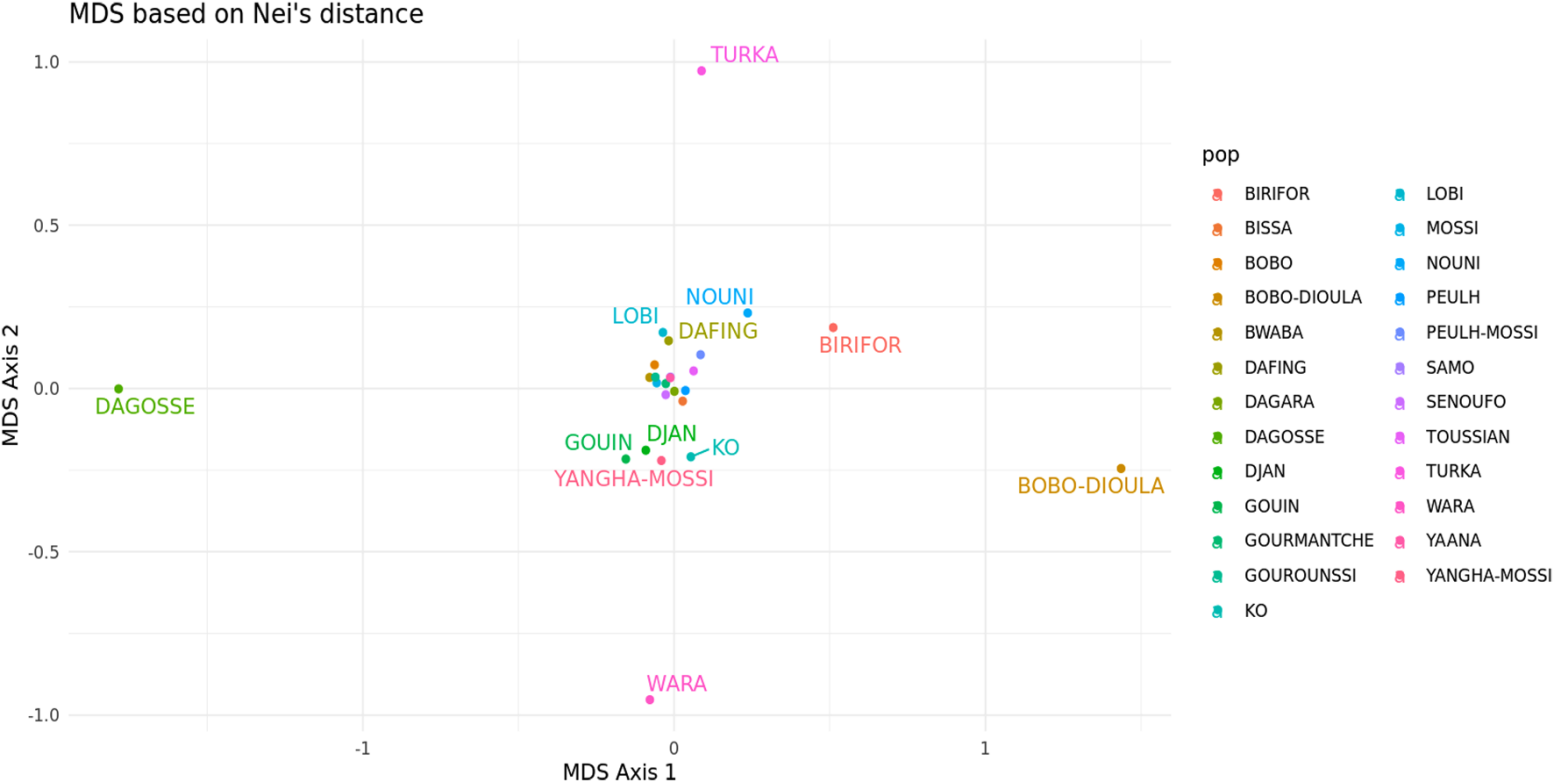
MDS based on Nei's genetic distance in Burkina Faso ethnic groups (19-XSTR loci). MDS generated using STR Analysis for Forensics software (STRAF) version 2.1.5 software^34^.

The pooled dataset of males and females was run on STATSX software to assess the genetic distance between the different ethnic groups involved in our study and to do Multidimensional Scaling (MDS) analyses. Figure 4 shows the genetic distances between the participants in this study. The results showed a main cluster in the middle of the MDS plot formed by most of the ethnic groups in Burkina Faso. The Turka, Bobo-Dioula, Wara, and Dagosse ethnic groups were not associated with the main cluster in the BF.

## Discussion

### aSTRs

Forensic parameters and genetic structure analyses based on 21 autosomal STR loci showed that this allele frequency database can be useful for forensic casework and population genetic studies of the BF population. At the time of writing the article, very few autosomal STR datasets existed involving African populations, and among the available published datasets, the set of loci was different from that used in our study. Despite this observation, we succeeded in gathering published data from Africa using a set of 14 common loci (D19S433, D5S818, D21S11, D18S51, D3S1358, D13S317, D7S820, D16S539, CSF1PO, D8S1179, TPOX, TH01, D2S1338, and FGA) for comparison.

In comparison with previously published data on the West African population, the CPE value found in this study (CPE for “duos” was 0.999999837, while the CPE value for “trios” was 0.999930699) was higher than that reported by Li et al.^21^ in Sierra Leone population, where the CPE for “Duos” was 0.99999343. However, the CPE for “trios’’ were lower than that found in the same study of Li et al.^21^ where the CPE was 0.9999999895. We observed that our CPE value was lower than that of CPE: 0.999999999 and 0.9999999632 found in neighbouring country population names Ghana^22^ and Nigerian population^23^, respectively.

The MDS based on Nei’s distance carried out using 14 STR allele frequencies (Table S5) was used to display Nei's distance between the different populations involved in this study (Table S4). BF populations clustered together with other west African populations in a large cluster. We found that the BF-Mande population was closer to the Nigerian-Haoussa subpopulation groups than the neighboring country Ghana-Akan^22^ (Fig. 3). In contrast, the BF-Gur subpopulation was closer to the Nigeria-Yoruba, Nigeria-Igbo^23^, South Africa-SEB^24^, and Sierra Leone^21^ subpopulations. African populations that showed very distinct genetic ancestries in previous studies such as Khoe, San, Coloured in South Africa^24^ and Kenya-Cushitic^25,26^ were separated from the west African main cluster, including the BF Mande and GUR populations. Non-African populations such as Thaïs, Chechen in Jordan^27^ and Tijuana in Mexico^28^ were also separated from the African populations. The BF ethnolinguistic grouped and labelled “Others” were particularly separate from the west African cluster around BF-MANDE and BF-GUR. This suggests that future studies should incorporate whole-genome data from smaller populations within BF to better understand the genetic landscape of West African populations.

Among the 21 aSTR typed in this study, loci D13S317, D7S820, and TPOX showed the lowest number of alleles, with eight alleles. The remaining set of loci used in this study showed high forensic efficiency values for the BF population sample, supporting the application of these loci for forensic analyses in BF.

### X-STRs

In general, genetic relationship comparisons between target populations based on the same microsatellites (STRs) are performed using an input file of either the genotypes or allelic frequencies. To perform such a comparison between the populations involved in this study and other populations based on the same 19 X-STR loci extracted from the literature, very few studies were available at the time of writing this article.

**Figure 5:**
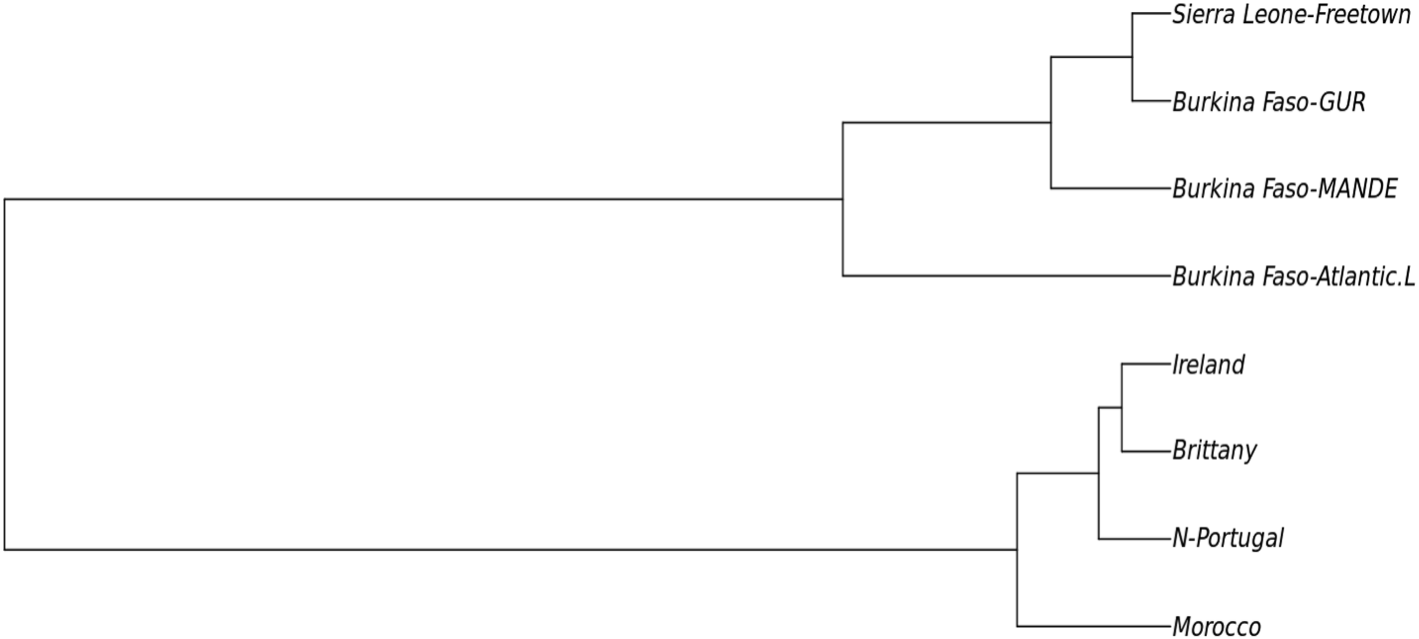
Phylogenetic relationship between the population from Burkina Faso and other published populations based on Nei’s genetic distance (generated using STR Analysis for Forensics software (STRAF) version 2.1.5 software^34^).

**Figure 6:**
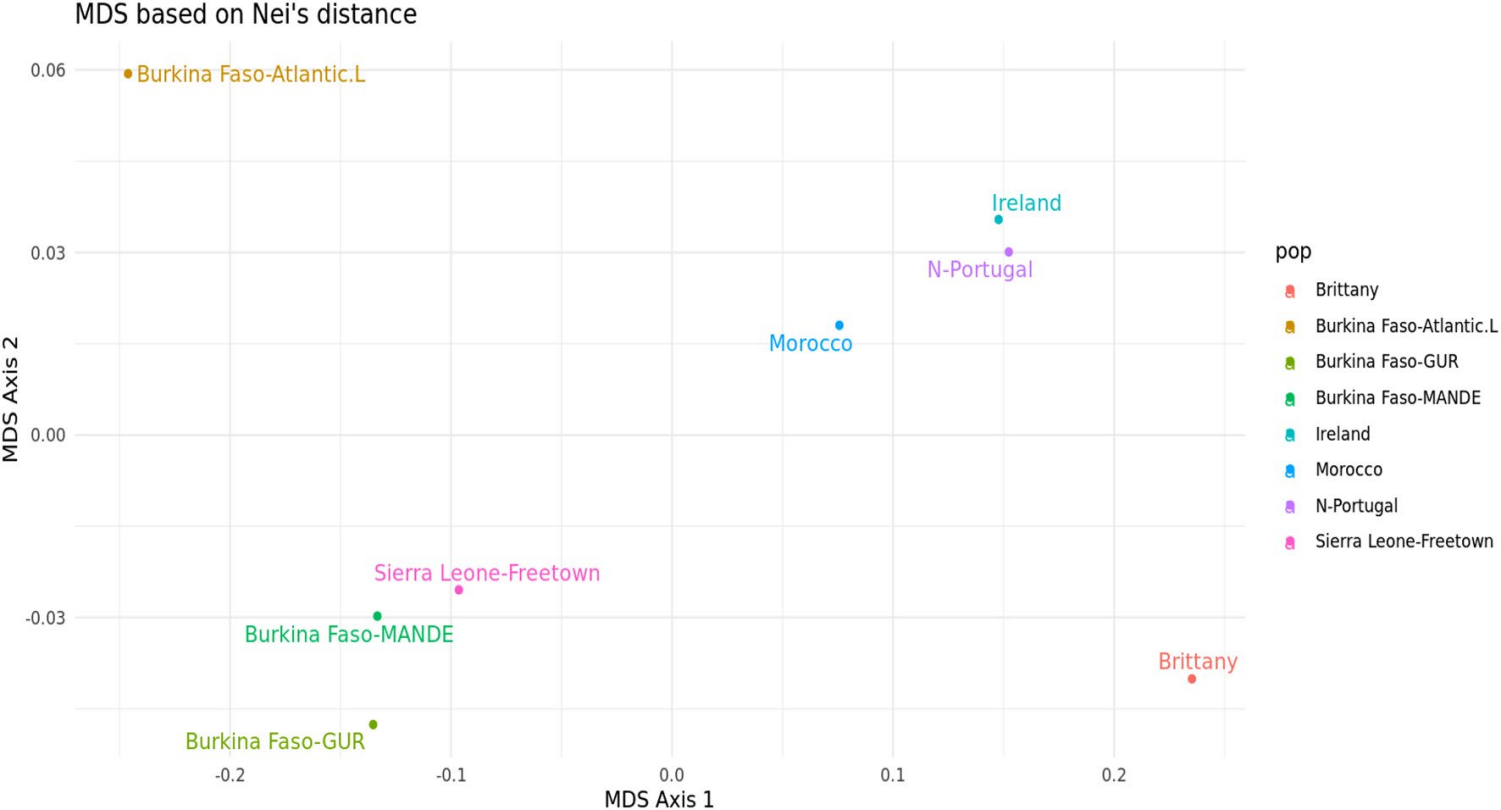
MDS based on Nei's genetic distance between the Burkina Faso populations and other published populations (generated using STR analysis for forensics software (STRAF) version 2.1.5 software^34^).

Despite the limitations of datasets available for African populations, we were able to compare the genetic relationship between the BF population and some published studies based on 10 X-STR loci (we targeted the largest number of loci in common between the published studies to provide a reliable genetic comparison between populations). Figure 5 displays the phylogenetic tree of the included populations and Table S11 shows the pairwise F_ST_ genetic distance values between populations. The input file used in the STRAF online software was created based on the genotype profiles of the individuals involved in the studies. A total of 1356 individuals belonging to seven populations from different countries were included in this study for comparison. The target population was Sierra Leone individuals living in the capital city of Freetown, West Africa, as reported by Lin et al.^20^. Populations of the Atlantic coast in Europe (Britany, Ireland, and Northern Portugal) and northwestern Africa (Morocco), as described by Prietro et al.^29^, were included in this genetic distance comparison. In the MDS analyses, Fig. 6, clusters were formed between the BF populations of Mande and Gur speakers (p = 0.0030) and the population of Freetown, the capital city of Sierra Leone. Interestingly, BF Atlantic speakers grouped separately towards the top of the MDS 2 axis.

MDS plots provide an intuitive two-dimensional representation of complex genetic relationships between several populations. Populations who are genetically more related will appear closer to each other in the two-dimensional space. Genetic comparisons between the BF population (FST values are listed in Table S11) and the reference population showed that the BF Atlantic language speakers were genetically less related to Mande and Gur speakers, than these two populations to each other and to the Sierra Leone population (Fig. 6). This can be explained by the fact that Atlantic language speakers, represented by the ethnic group Fula/Peulh, live in a closed community and tend to have intermarriage within this community^30^. Therefore, their genetic background are different from other groups.

## Conclusion

This study on forensic autosomal and gonosomal short tandem repeat marker reference databases is a pioneer study for BF population genetic data and forensic genetic investigations. This work supports previous studies on Y-STR for the BF population by providing reliable forensic parameters that could be used in forensic casework, especially in deficient paternity casework in the context of BF. The X-STR panel also has benefits in providing information about population structure and substructure that shape the multicultural population living in Africa, particularly in BF. Through the results obtained in this study, which demonstrated that the loci used in the MICROREADER 19X Direct ID System kit and MICROREADER 21 ID System kit were highly polymorphic and informative, we conclude that these commercial kits are suitable for the BF population.

## Materials and methods

### Ethical requirements and sample collection

This study followed the recommendations for the publication of population data and the guidelines provided by the Declaration of Helsinki^31,32^. This research was approved by the BF National Ethics Committee, and ethical approval was issued by the BF Health Research Ethics Committee (deliberation No: 2020-01-004). Each participant voluntarily signed a written informed consent form before providing biological samples. Saliva swab samples were then collected from all the participants. Self-declared-unrelated, and healthy individuals living in the capital city of Ouagadougou were recruited.

For the aSTR analysis, 396 donors (272 males and 124 females) were included. All participants belonging (29/63) to ethnolinguistic groups gave their ethnic group names in writing after the study aims and procedures were explained carefully. Overall, the volunteers spoke languages belonging to the Niger-Congo language phylum. They are either from Mande or Gur family except few ethnic’s groups labelled “Others”. The ethnic groups involved in this study and the number of individuals (in brackets) are listed in this section. For the MANDE language family, the 54/396 individuals recorded were as follow: Bissa (10), Bobo (14), Dafing (6), Dioula (2) and Samo (22). The GUR family comprised 331/396 individuals. Among the GUR family there were the following ethnic groups: Birifor (02), Bwaba (21), Dagara (13), Dagosse (01), Djan (01), Gan (01), Gouin (04), Gourmatche (22), Gourounssi (27), Karaboro (02), Lobi (11), Mossi (203), Nouni (01), Senoufo (08), Sissala (01), Tiefo (01), Toussian (04), Turka (02), Yaana (05), and Zoaga (01).

For the XSTR analysis, a total of 292 unrelated people based on self-declaration from different ethnic groups among the BF population were sampled. Among the 292 volunteers (123 females and 169 males) in this study, 247 people belonged to the Gur language family. Inside the Gur language family, there were: Birifor (01), Bwaba (12), Dagara (09), Dagosse (01), Djan (01), Gouin (04), Gourmantche (21), Gourounssi (21), Ko (01), Lobi (08), Mossi (155), Nouni (01), Senoufo (05), Toussian (04), Turka (01), Wara (01), Yaana (04), and Yangha-Mossi (01). Within the dataset, 40 individuals belonged to the Mande language family, distributed as follows: Bissa (08), Bobo (13), Bobo-dioula (01), Dafing (05), and Samo (13). The last language family reported in our dataset was the Atlantic language, with mainly the Peulh or Fula ethnic group (05).

Overall, for statistical reasons, the samples of language families not reaching 10 individuals per ethnic group, have been grouped into a group labelled “others”. All the details concerning the languages of BF are available through the following link: http://sil-burkina.org/en/content/languages-burkina-faso.

### DNA extraction from oral swabs

Saliva swab samples were obtained from all volunteers recruited for this study. Genomic DNA was extracted using a HiPure Universal DNA kit (Magen), according to the manufacturer’s protocol.

### Quality control

A Nanodrop 1000 spectrophotometer (Thermo Fisher Scientific Inc. Wilmington, DE, USA) was used to assess DNA purity and concentration before genotyping. Positive and negative controls were used to evaluate the reliability of results during genotyping. The standard DNA 9947A positive control was used as the positive control, which was provided with the MICROREADER 19X Direct ID System kit and also with the MICROREADER 21 ID System kit. De-ionised water for the amplification reaction (ddH2O) was used as the negative control.

### Autosomal STRs and X-STRs genotyping

The MICROREADER 21 ID (V3.1) System kit and MICROREADER 19X Direct ID System kit (MICROREADER Genetics, Zhongguancun Science and Technology Park, Haidian District, Beijing, China) were used to amplify the autosomal STR and X-STR loci, respectively, according to the manufacturer’s instructions. DNA was amplified using the GeneAmp PCR System 9700 (Applied Biosystems, USA) following the manufacturer’s instructions.

The PCR products were separated in both cases (aSTR and X-STR) by capillary electrophoresis on an ABI 3130XL Genetic Analyser (Applied Biosystems, USA). Allele identification was performed using GeneMapper IDX V1.5 (Applied Biosystems) using the allelic ladders provided with the MICROREADER 21 ID (V3.1) System kit and the MICROREADER 19X Direct ID System kit^18^. The 21 ID System kit and 19X Direct ID System kit alleles were designated according to the International Society for Forensic Genetics (ISFG) recommendations^33^. The MICROREADER 21 ID System kit uses 6-colors fluorescent markers and multiple amplifications to detect the following 21 aSTR loci: D19S433, D5S818, D21S11, D18S51, D6S1043, AMEL, D3S1358, D13S317, D7S820, D16S539, CSF1PO, Penta D, D2S441, vWA, D8S1179, TPOX, Penta E, TH01, D12S391, D2S1338, and FGA. The MICROREADER 19X Direct ID System kit also uses 6-color fluorescent markers and multiple amplifications to detect a panel of STRs, and the following 19 X-STR loci were identified: DXS6795, DXS6803, DXS6807, DXS9907, DXS7423, GATA172D05, DXS101, DXS9902, DXS7133, DXS6810, GATA31E08, DXS6800, DXS981, DXS10162, DXS6809, GATA165B12, DXS10079, DXS10135, and HPRTB.

### Statistical analysis for aSTRs and X-STRs genotype data

For aSTR dataset analysis, forensic-relevant parameters, such as gene diversity (GD), polymorphism information content (PIC), matching probability (MP), power of discrimination (PD), and allele frequencies, were generated using STR Analysis for Forensics software (STRAF) version 2.1.5 software^34^. The observed heterozygosity (Ho), expected heterozygosity (He), probability values of the Hardy–Weinberg equilibrium exact test (pHWE), and linkage disequilibrium (LD) were calculated using ARLEQUIN v3.5 software^35^. Paternity test parameters, such as the power of exclusion for trios (PE trios) and Typical Paternity Index (TPI), were estimated using an Excel sheet applying the formula described by Marshall et al.^36^. A neighbour-joining (NJ) tree based on pairwise F_ST_ values was constructed using MEGA 7.0^37^, based on the F_ST_ values generated by STRAF. The Interactive Tree of Life (ITOL) software (https://itol.embl.de)^38^ was used to display, manipulate, and annotate the phylogenetic trees.

For the analyses of the X-STR dataset, we estimated the power of exclusion (PE), homozygosity (h), heterozygosity (HET), power of discrimination in females (PDF), and males (PDM), as well as the mean paternity exclusion chance (MEC), in specific cases (mother, daughter, paternal grandmother) in which the paternal grandmother was investigated instead of the alleged father. This value was computed according to the method described by Krüger et al.^39^. In standard trio cases involving daughters, this value was computed according to Kishida et al.^40^ MEC is defined as the mean probability that an individual has a genotype that differs from that of randomly chosen individuals in a population^41^. Allele frequencies, haplotype diversity (HD), gene diversity (GD), and Polymorphism Information Content (PIC) were computed using the software named StatsX (Statistics for X-STR) v2.0^41^. The software STATX v2.0 and the online calculation tool ChrX-STR.org 2.0, were used to compute the following forensic parameters: PDF, PDM, MECKrüger, MECKishida, MECDesmarais, and MECDesmarais Duo.

Linkage disequilibrium (LD) testing between all pairs of the 19 loci in females, as well as the Hardy–Weinberg equilibrium (HWE) test, were performed on female samples using ARLEQUIN v3.5^35^. LD and HWE calculations were applied after exporting the data file in the ARLEQUIN format, using STATX v2.0. For male samples, the exact test of LG between all pairs of the 19 X-STR loci was performed using ARLEQUIN v3.5.

### Supplementary Information

Supplementary Tables.

## Data Availability

The datasets used and/or analysed during the current study are available from the corresponding author upon reasonable request.
